# Topological vulnerability of power grids to disasters: Bounds, adversarial attacks and reinforcement

**DOI:** 10.1371/journal.pone.0204815

**Published:** 2018-10-12

**Authors:** Deepjyoti Deka, Sriram Vishwanath, Ross Baldick

**Affiliations:** 1 Center for Non-Linear Studies, Los Alamos National Laboratory, Los Alamos, United States of America; 2 Department of Electrical and Computer Engineering, The University of Texas at Austin, Austin, United States of America; University of Padua, ITALY

## Abstract

Natural disasters like hurricanes, floods or earthquakes can damage power grid devices and create cascading blackouts and islands. The nature of failure propagation and extent of damage, among other factors, is dependent on the structural features of the grid, that are distinct from that of random networks. This paper analyzes the structural vulnerability of real power grids to impending disasters and presents intuitive graphical metrics to quantify the extent of topological damage. We develop two improved graph eigen-value based bounds on the grid vulnerability. Further we study adversarial attacks aimed at weakening the grid’s structural robustness and present three combinatorial algorithms to determine the optimal topological attack. Simulations on power grid networks and comparison with existing work show the improvements of the proposed measures and attack schemes.

## Introduction

The structure of the power grid is an important feature that affects the delivery of electricity [1]. From an economic perspective, the capacity of transmission lines and the graphical properties (whether tree-like or loopy) affect the locational marginal electricity prices as well as the convergence of Optimal power flow algorithms [2]. At a faster time-scale, structure of the grid affects controllability of ambient fluctuations [3]. From a reliability perspective, the grid structure is one of the factors that influence the extent of damage following a natural or man-made disaster. In particular, it affects the propagation of failures and the formation of islands. Over the years, natural disasters like earthquakes, floods, hurricanes have caused extensive power outages due to damage of grid equipment and loss of network connectivity [4, 5]. Such disasters also directly and indirectly affect other co-located and interdependent networks like the transportation and communication infrastructure. Thus there is a greater need to quantify the effect of the grid structure on failure propagation in the grid following a natural disaster and to incorporate the insights gained into transmission planning techniques to improve grid resilience.

There is existing work that studies the impact of different grid features including structure on grid reliability, under different regimes of operation. Reference [6] describes a DC model for the propagation of equipment (nodes and links) failures in cascading outages in the power grid. Here, the propagation process begins with an initial failure of a network node or link which leads to a redistributing of the power flows for optimal dispatch. Subsequently in [7], the authors incorporate recovery mechanism for the tripped transmission lines into the failure model and show that the size of blackout has a power-law distribution. Reference [8] analyzes the problem of finding the optimal *k* lines in the grid that can be used for interdiction in the grid to create failures. Power flow based analysis has been done to analyze geographically correlated failures in [9]. Similarly, an interdiction based analysis on grid resilience considering short term impacts is discussed in [10]. These models utilize laws governing power flows and generator dispatch to solve optimization problems such as Optimal Power Flow (OPF). Such dispatch decisions are effective in the range of 5 − 15 minutes in realistic power grid operations. At time-scales below the minute level, voltage and frequency fluctuations represented by swing equations affect the stability of the grid. In that regime, control-theoretic measures [3, 11, 12] have been used to analyze grid resilience and suggest improvements to prevent damage. Indeed inclusion of accurate models for generator dynamics, load response and system operator’s control efforts into the analysis improve the characterization of the physics of failure propagation and enable an accurate study. Such analysis, however, seldom leads to a closed form expression of a metric to quantify the resilience of the power grid to failures, one of the objectives of this paper. Moreover perspective gained from an detailed optimization based quantification of grid vulnerability may not be transferable from one network to another. Further it needs to be mentioned that power grid dynamics in the regime of an extreme event like cascading outage is highly non-linear and may not be accurately represented by dynamical equations approximated for small ambient fluctuations.

In a separate line of work, efforts have been made to study probabilistic failure propagation in power grids and related networks using techniques from percolation theory and random graph theory. In this approach, failures based on connectivity as against power flows are studied. Initial failures induced by external events are supposed to propagate probabilistically from source to neighboring nodes and edges in the grid graph. The final state will often include greater number of failures than the initial state and efforts are made to study the effect of the grid structure in influencing the spread. References [13, 14] study the effect of removing nodes and edges from power grid graphs based on their centrality and degree measures and their effect on network connectivity. The authors of [15, 16] analyze the propagation of structural failures in complex random networks based on similar neighborhood propagation rules. This approach has been extended to study interdependent networks failures as well [17–20] where nodes of two different networks may depend on one another for survivability. Similarly, references [21, 22] have analyzed node and edge percolation based techniques to understand failure propagation in random graphs generated by stochastic geometry. An interaction graph based model is presented in [23] where power flow based cascading information is used to study the inter-nodal dependencies on cascades. For mitigation of cascading failures, a capacity sharing protections scheme is proposed in [24]. A good review of works pertaining to grid resilience to natural disasters can be found in [25] and [26].

It is worth mentioning that parallel analytical graph-theoretic techniques are also used in studying social and biological networks for information dissemination, spread of viruses as well as synchronization of dynamic processes [27–29]. Existing work [1, 30–34] has demonstrated that the structure of finite-sized real power grids create deviations in observed parameters from those predicted in random graphs. For example, sharp breakdown thresholds that emerge in analysis of topological failures on random graphs [17, 19] are seldom observed in simulations of failures on real grids and IEEE test cases [35]. Further, structural reinforcements derived from random graph analysis [19] include fractional network changes (relative to size of the entire grid) where effects of nodes of similar degree or connectivity are not distinguished. Such changes are not easily extended to determine finite (optimal *k*) network upgrades as they do not use the exact topology of the grid that is available with the grid controller.

In this work, we focus on power grid failures induced by large natural disasters like hurricanes and earthquakes that create disconnected islands in the grid. Our work pertains to graph-theoretic analysis of connectivity based failure models in power grids. This is similar to mentioned prior work on probabilistic failures in power grids but has a key difference. We use knowledge of the finite sized power grid graphs in our analysis and show that this leads to non-trivial results and improved understanding of network vulnerabilities. For grids affected by a disaster, we study the post-disaster network and present justification for using the size of the largest connected component as a valid metric for the functioning grid. We then extend probabilistic analysis of grid failures to known real grid graphs and popular IEEE test cases and determine computable graphical parameters (eg. eigenvalues of the grid adjacency matrix) that can be used to quantify grid robustness. It is noteworthy that these bounds are not realized from random graph constructions in prior work that do not consider the known finite structure of the grid. More importantly, we also present a novel construction of a modified graph based on the original grid graph that can be used to develop improved bounds on the extent of damage created by the disaster. The efficacy of the graphical parameter based bounds and soft thresholds are demonstrated by simulations of failures on publicly available grid data-sets.

The final contribution of the paper is in determining grid components that affect its vulnerability. We use the graphical metrics of grid resilience mentioned above to identify *k* critical transmission lines (graph edges) that maximally affect the grid robustness. In particular, we study removal of transmission lines by an adversary that aims to maximize the expected damage to network connectivity following a natural disaster. As this problem is NP-hard in general, we present three novel approximate algorithms to determine the optimal edges in the adversary’s target set. The first two algorithms are based on perturbation based analysis of eigen-values of the grid adjacency matrix while the third algorithm is based on greedy trace minimization of a higher power of the adjacency matrix. Note that all three developed approaches rely on the global properties of the finite-sized grid structure. Crucially, such algorithms do not emerge from random graph based prior work where nodal degrees and centrality measures may be used to determine vulnerable locations. The performance of our algorithms for attack design in reducing grid resilience is demonstrated through simulations. Further comparison from other random graph based topological techniques in literature [14] are presented to elucidate the benefits derived from our approach. From the system operator or grid controller’s perspective, these algorithms can be used to determine the critical lines that need to be protected to build resilience before any impending natural disaster.

To summarize, we present a framework to *analytically quantify the ‘topological’ resilience of real power grids to natural disasters*, and develop *novel algorithms to determine critical transmission lines that can prevent adversarial deterioration*. The rest of the paper is organized as follows. In the next section, we develop our intuitive graph theoretic quantification of network failure. Next, in Section 1 we analyze failures in actual grid graphs (non-random) and present our preliminary bound on the threshold of network breakdown. We proceed to present our novel modified graph construction to develop improved bounds on the threshold and size of the network damage in Section 1. We also discuss simulation results of developed bounds on IEEE test cases and real power grids. In Section 1, we study adversarial attacks on transmission lines aimed at weakening grid resilience to natural disasters and present three approximate methods to determine the critical transmission lines. Simulation results on our designed algorithms and comparison with existing work highlight the benefits of our approach in Section 1. Limitations of our current approach and directions of improvements are discussed in Section. Finally, we discuss the insights gained and prospective future work in Section.

## Failure model in power grids

We begin by describing the power grid and the topological failure model considered in this paper.

### Network Model

**Fig 1:**
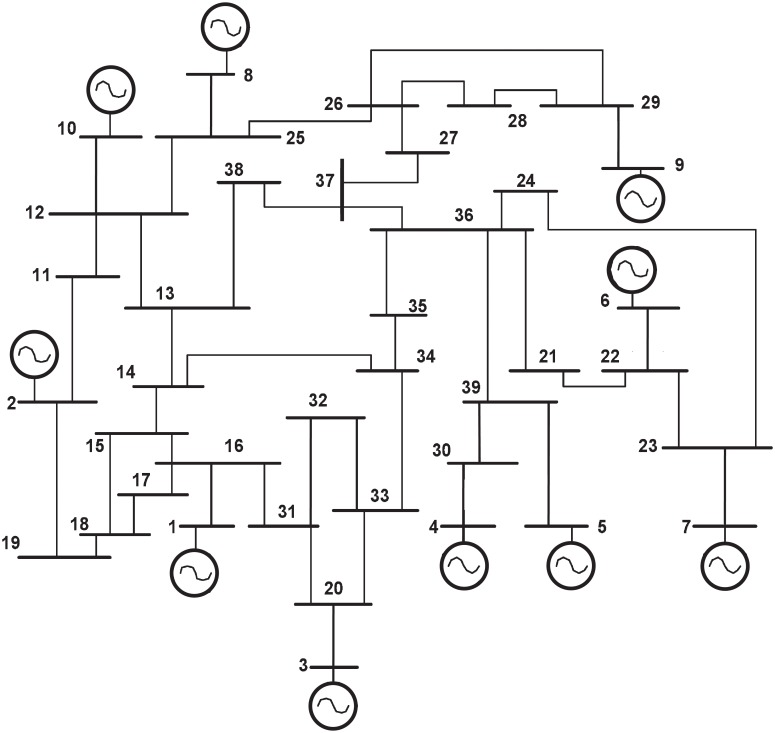
IEEE 39-bus test system [35].

We denote the grid by a graph G=(V,E), where sets *V* and *E* represent the nodes/buses and the undirected edges/lines respectively. Let the total number of buses in the system be *N*. We denote the adjacency matrix of the graph G by $A_{G}$ that is assumed to be known and not generated by a probabilistic model. Each edge (*ij*) in *E* is represented by a value of 1 for $A_{G}\left(i,j\right)$ and $A_{G}\left(j,i\right)$ in the binary adjacency matrix. As an example, the IEEE 39 bus test system [35] is given in Fig 1. We assume nodes in the grid to have generation resources that may be provided by renewables (solar, wind etc.) or by conventional resources. Further, under normal operating conditions, the lines are assumed to have sufficient transmission capacity to transfer power from one part of the network to another. This is done as the objective of the paper is to study only topological (not electrical) features of the grid and their effects on grid resilience.)

### Failure Model

**Fig 2:**
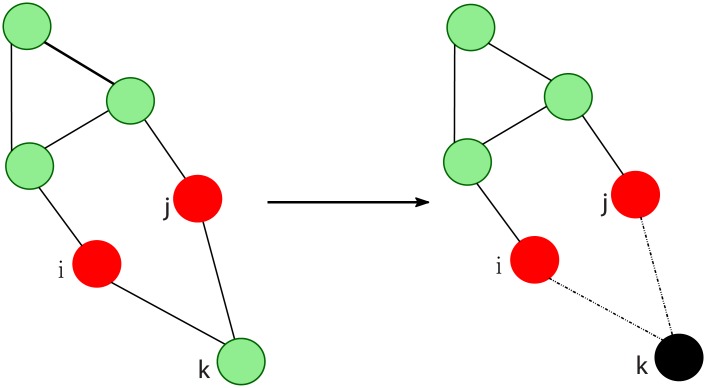
Primary failures, colored red, at nodes *i* and *k*. Secondary failure, colored black, at node *j* as it is disconnected from the network due to failures at its neighboring nodes. The surviving nodes are colored green.

As described in the Introduction, we consider a natural disaster that causes a probabilistic failure on all nodes in the system, with independent *initial probability of failure* denoted by *p*_0_. The initial failure probability *p*_0_ depends on the nature of the natural disaster (Eg. earthquake scale, wind speed of hurricane etc.) as well as geography (Eg. topography of the land). Such probabilities are computed by agencies like the National Hurricane Center and used to predict the scale of damage and help in planning for evacuation strategies [36]. For small grids with small or moderate geographical coverage, the assumption of identical initial probability of failure is valid. For a grid in a large area with diverge topographical conditions, the initial probability of failure will vary from node to node. In this work, we only consider cases where the initial probability of failure on all nodes is the same. The case with different initial failure probabilities for nodes is briefly discussed in comments and will be a subject of our following work. Under the failure model, the nodes can fail in two ways. One, nodes may fail under the initial failure probability—we term such failures as *‘primary’ failures*. Second, nodes fail as they get disconnected from the rest of the network due to failures at all neighboring nodes. Such failures are termed as *‘secondary’ failures*. Both these kind of failures are depicted in Fig 2. As described in subsequent discussion, we consider a node to be disconnected if it is separated from the largest connected component in the surviving network. Note that the chance of overall failure of a node depends on both the initial probability of failure *p*_0_ and the probability of being disconnected which in turn depends on the network structure. Next we describe our measure of network damage following the natural disaster.

### Measure of network damage

We assume that transmission line capacities are sufficient to satisfy all load in the system provided enough generating resources are online and connected. Thus in the post event network, loads are not served only if they are not connected to sufficient generation resources. In this work we restrict our discussion to strictly topological measures of network damage. In Section 1 we discuss possible ways of integrating other schemes of network damage into our models.

Let *N*_*s*_ denote the size of the largest connected component of surviving nodes after the disaster. We consider *N* − *N*_*s*_ to be the *measure of network damage* caused by the natural disaster in this paper. It is worth mentioning that outside of the largest component, smaller groups of nodes can be functional as well. However, we select *N* − *N*_*s*_ as the measure of network damage as it has a key characteristic as described next.

Let $\Delta_{i}^{P}$ denote the net power (generating capacity minus load) of node *i* in the network, where nodal generating capacity and load are both random variables. Note that net power may be positive or negative. We model each $\Delta_{i}^{P}$ by an independent Gaussian random variable with mean *μ* ≥ 0 and variance *σ*^2^, as commonly used in literature [37, 38]. The variance of the Gaussian random variable reflects the uncertainty with nodal injections.

If node *i* is disconnected from the rest of the network, the probability $q_{0}^{i}$ that its local load is served is then given by:

$$\begin{array}{cc} & q_{0}^{i}=P\left(\Delta_{i}^{P}\geq 0\right)=\int_{0}^{\infty }\frac{1}{\sqrt{2\pi \sigma^{2}}}e^{-{\left(x-\mu \right)}^{2}/\sigma^{2}}dx\\ \Rightarrow & q_{0}^{i}=.5+\int_{0}^{\mu }\frac{1}{\sqrt{2\pi \sigma^{2}}}e^{-{\left(x-\mu \right)}^{2}/\sigma^{2}}dx\;\text{as}\;\mu \geq 0 \end{array}$$ (1)

On the other-hand, if node *i* is connected to a group of *N*_*k*_ nodes, the probability $q_{N_{k}}^{i}$ that node *i* satisfies its load is given by:

$$\begin{array}{cc} q_{N_{k}}^{i} & =\mathbb{P}\left(\sum_{i=1}^{N_{k}}\Delta_{i}^{P}\geq 0\right)=\mathbb{P}\left(\sum_{i=1}^{N_{k}}\Delta_{i}^{P}/N_{k}\geq 0\right)\\ & =.5+\int_{0}^{\mu }\frac{1}{\sqrt{2\pi \sigma^{2}/N_{k}}}e^{-\frac{{\left(x-\mu \right)}^{2}}{\sigma^{2}/N_{k}}}dx \end{array}$$ (2)

where Eq (2) follows from the fact that $\sum_{i=1}^{N_{k}}\Delta_{i}^{P}/N_{k}$ is a Gaussian(*μ*, *σ*^2^/*N*_*k*_) random variable. Note that its variance decreases with increase in *N*_*k*_, the size of the connected set that node *i* belongs to. For 1 ≤ *N*_*k*_ ≤ *N*_*s*_, using properties of the exponential function it follows that

$$\begin{array}{cc} & \int_{0}^{\mu }\frac{1}{\sqrt{2\pi \sigma^{2}/N_{k}}}e^{-\frac{{\left(x-\mu \right)}^{2}}{\sigma^{2}/N_{k}}}dx\leq \int_{0}^{\mu }\frac{1}{\sqrt{2\pi \sigma^{2}/N_{s}}}e^{-\frac{{\left(x-\mu \right)}^{2}}{\sigma^{2}/N_{s}}}dx \end{array}$$ (3)

Using Eqs (1), (2) and (3), we have $q_{1}^{i}\leq q_{N_{k}}^{i}\leq q_{N_{s}}^{i}$.

Thus, *the probability of a nodal load being served is maximum when it belongs to the largest set of connected nodes*. This justifies our usage of *N*_*s*_ (size of the largest connected component of surviving nodes) to quantify the functional network and correspondingly of *N* − *N*_*s*_ to measure the scale of network damage. Note that prior literature includes the use of the largest connected component in simulation based studies [39] of grid resilience. However the motivation behind its usage in power grids is not clearly stated or justified. In the next section, considering the largest component as the surviving network, we analyze the effects of network structure on the extent of failures and determine a preliminary bound on the probability of initial failure *p*_0_ beyond which the network fragments.

## Failure analysis and preliminary bound

As shown in Fig 2, we consider primary failures and secondary failures in the grid. The overall survival probability of each node depends on the absence of both failure cases and thus depends on the probability of initial failure and network structure. Let λ^*V*^(*i*) denote the survival probability of node *i* in set *V*. Due to the neighborhood based dependence of secondary failures, λ^*V*^(*i*) satisfies the relation

$$\lambda^{V}\left(i\right)=\left(1-p_{0}\right)P\left[\underset{j:(ij)\in E}{⋃}\left\{\text{node}\;j\;\text{survives}\right\}\right]$$ (4)

$$\Rightarrow \;\lambda^{V}\left(i\right)\leq \left(1-p_{0}\right)\sum_{j:(ij)\in E}\lambda^{V}\left(j\right)$$ (5)

$$\Rightarrow \;\lambda^{V}\leq \left(1-p_{0}\right)A_{G}\lambda^{V}\;\left(\text{in}\;\text{vector}\;\text{form}\right)$$ (6)

Note that 1 − *p*_0_ is the probability of *i* surviving the primary failure, while the rest of the terms in Eq (4) denote the probability of at least one surviving neighbor node that connects it to the largest component and prevents secondary failure. Here Eq (5) follows from the Union Bound [40] for probabilities. Note that the union bound is not replaceable by an equality for grid graphs as failures at different nodes are correlated due to sharing common paths for connectivity. This is distinct from prior work in random graph models [17] where equality is assumed based on locally tree-like structural assumptions on the grid structure. In a related setting with varying initial probabilities of failures for different nodes ($p_{0}^{i}$ for node *i*), we modify Eq (6) to compute the overall probability of failure. In particular, (1 − *p*_0_) on the right side of Eq (6) is replaced by a diagonal matrix with $\left(1-p_{0}^{i}\right)$ as the *i*^*th*^ diagonal entry. In the remaining sections, we limit our discussion to a constant $p_{0}^{i}=p_{0}\forall i$ as we are interested in deriving thresholds based on it. Note that one can derive an upper bound on the value of λ^*V*^ by computing fixed point iterations of relation (6) till the values converge. Using that we have the following upper bound on the initial probability of random failures (*p*_0_) beyond which the grid fragments.

**Theorem 1** *Let* $\beta_{A_{G}}$ *be the largest eigenvalue of the adjacency matrix* $A_{G}$. *If* $p_{0}>1-1/\beta_{A_{G}}$, *the grid fragments following the natural disaster*.

The proof follows immediately from the fact that if $\left(1-p_{0}\right)\beta_{A_{G}}<1$, then λ^*V*^ → **0**. Thus, we have an **upper bound** on *p*_0_. Note that this is indeed a bound and not an exact threshold due to correlated final probability of failures at different nodes. Further the current formulation does not specify the extent of damage in the region $p_{0}<1-1/\beta_{A_{G}}$. In the next section, we present a novel modified graph construction based on the true grid graph that overcomes this and helps generate tighter bounds.

## Modified graph for improved bounds

**Fig 3:**
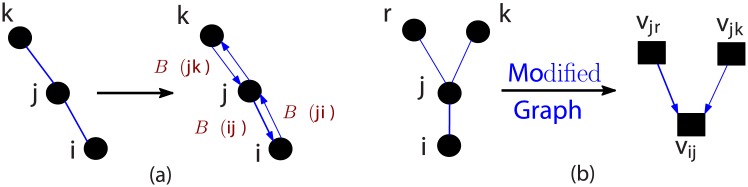
(a) Each undirected edge results in two survival probabilities, one in each direction. (b) Modified graph $G^{E}$ formation from G.

In our failure model, the survival of any node depends on the existence of edges that connect it to the largest connected component. We now analyze a node’s survivability by modeling the probability of its connectivity through operational edges in the grid graph. This is done by construction of a *‘modified’* graph based on the true grid graph. To motivate this approach better, consider node *i* and its neighbor *j* in the grid as shown in Fig 3(a). Let *B*^*E*^(*ij*) be the event that *i* is connected to the surviving nodes in the largest component through edge (*ij*). Let the probability of *B*^*E*^(*ij*) be denoted by λ^*E*^(*ij*). Similarly, such events can be defined for every neighboring node of node *i*. Note that node *i*’s survivability requires at least one of these events to be true. Thus, the probability of node *i* surviving is given by:

$$\begin{array}{c} \lambda^{V}\left(i\right)=\left(1-p_{0}\right)P[\underset{j:(ij)\in E}{⋃}\left[B^{E}\left(ij\right)\right]\;\text{where}\;P\left[B^{E}\left(ij\right)\right]=\lambda^{E}\left(ij\right) \end{array}$$ (7)

Here, the (1 − *p*_0_) term arises from the probability of node *i* surviving a primary failure, while the remaining terms correspond to the survivability due to at least one connecting edge. In a similar way, we define event *B*^*E*^(*ji*) of probability λ^*E*^(*ji*) for node *j* surviving through the edge with node *i*. This event is reciprocal to *B*^*E*^(*ij*). Thus, every edge gives rise to two probabilities of survival, one along each direction as shown in Fig 3(a). If nodes *i*, *j* and *k* are connected as shown in Fig 3(a), the event *B*^*E*^(*ij*) (node *i* surviving via edge (*ij*)) depends on node *j* not failing initially and events *B*^*E*^(*jk*) (*j* surviving through edge (*jk*)) for all neighbors *k* of node *j*. In terms of their probabilities, we write this relation mathematically ∀*i*, *j* such that (*ij*) ∈ *E* as

$$\begin{array}{cc} & \lambda^{E}\left(ij\right)=\left(1-p_{0}\right)P\left[\underset{k:(jk)\in E,k\neq i}{⋃}B^{E}\left(jk\right)\right]\\ \Rightarrow \; & \lambda^{E}\left(ij\right)\leq \left(1-p_{0}\right)\sum_{k:(jk)\in E,k\neq i}\lambda^{E}\left(jk\right)\;\;\text{(using}\;\text{Union}\;\text{Bound)} \end{array}$$ (8)

In Eq (8), the (1 − *p*_0_) comes from the fact that if node *j* fails initially, then node *i* cannot survive through edge (*ij*). Eq (8) is the motivation behind our construction of a **modified directed graph $G^{E}$** based on the power grid graph G. The objective of constructing this graph is to demonstrate the analysis of λ^*E*^(*ij*) that denote the probability of survival via paths to the largest connected component. As demonstrated, this leads to better estimates of the scope of network failures. The construction is depicted in the following steps:

Graph Construction for directed graph $G^{E}$ from grid G:
- Each edge (*ij*) in *E* gives rise to two nodes *v*_*ij*_ and *v*_*ji*_ in $G^{E}$, that represent orientations *i* → *j* and *j* → *i* in (*ij*).
- Two nodes *v*_*ij*_ and *v*_*kl*_ in $G^{E}$ are connected by an edge directed from *v*_*kl*_ towards *v*_*ij*_ if *k* = *j* and *l* ≠ *i* (see Fig 3(b)).

It is worth mentioning that $G^{E}$ is similar in structure to a line graph for grid graph G as nodes in $G^{E}$ arise from edges in G. However, there is a slight difference in that $G^{E}$ has lesser number of edges as a pair of nodes *v*_*ij*_ and *v*_*ji*_ are *not* neighbors in $G^{E}$, though they would be in a standard line graph construction. The number of nodes in $G^{E}$ is thus equal to twice the number of edges in G. The size of *A*_*E*_, the adjacency matrix of $G^{E}$, is 2|*E*| × 2|*E*|. Note that both graphs G and $G^{E}$ represent the same network but the later is able to model higher number of probabilities due to considering directional effects. In particular, each node *v*_*ij*_ in $G^{E}$ can be associated with probability of failure λ^*E*^(*ij*) that affects connectivity of node *i* through edge (*ij*) in the original graph G. Thus, we can write Eq (8) in a vector form using the adjacency matrix of the modified graph $G^{E}$ as follows:

$$\begin{array}{c} \lambda^{E}\leq \left(1-p_{0}\right)A_{E}\lambda^{E} \end{array}$$ (9)

Extending the analysis in the previous section to Eq (9), we get the following theorem that provides a **second upper bound** on the threshold on *p*_0_, beyond which the grid fragments.

**Theorem 2** *If* $\beta_{A_{E}}$ (*the largest eigenvalue of A*_*E*_) *satisfies* $p_{0}>1-1/\beta_{A_{E}}$, *then the grid disintegrates following the natural disaster*.

This follows as λ^*E*^ → **0** and thus λ^*V*^ → **0** when *p*_0_ is greater than the value in Theorem 2. We compare the first upper bound in Theorem 1 with this second upper bound for different IEEE test-cases and real grids and observe that in all cases the second upper bound is smaller in magnitude and hence provides an improved and tighter bound on the failure threshold. The comparisons are noted in simulation results later in this section. This improvement is due to a more nuanced modeling of the failure process through directed graph $G^{E}$ as compared to the original graph G.

We now use the modified graph $G^{E}$ and Eq (7) to analyze the extent of damage in the grid. In particular, we are interested in estimating the number of failures over the entire range of initial probability of failure *p*_0_, even below the bounds derived earlier. For this, we use the basic failure definition that states that a node fails eventually if it suffers an initial failure or if it gets disconnected from the largest component. In other words, the probability of *not* surviving (given by 1 − λ^*V*^(*i*) for node *i* in graph G) depends on the initial probability of failure and also on the probability that *none* of its edges connect it to the largest component. Let [*B*^*E*^(*ij*)]^*c*^ denote the event of node *i* *not surviving* through edge (*ij*). Using the notation in Eq (7), $P{\left[B^{E}\left(ij\right)\right]}^{c}=1-\lambda^{E}\left(ij\right)$. Mathematically, we have

$$1-\lambda^{V}\left(i\right)=p_{0}+\left(1-p_{0}\right)P\left[\underset{j:(ij)\in E}{⋂}{\left[B^{E}\left(ij\right)\right]}^{c}\right]$$ (10)

$$\leq p_{0}+\left(1-p_{0}\right)\min_{j:(ij)\in E}\left(1-P\left[B^{E}\left(ij\right)\right]\right)$$ (11)

$$\leq p_{0}+\left(1-p_{0}\right)\sum_{j:(ij)\in E}\frac{1-\lambda^{E}\left(ij\right)}{d_{i}}$$ (12)

where *d*_*i*_ is the number of neighbors of node *i* in G. Eq (10) follows from the failure definition where *p*_0_ denotes the initial failure probability for original node *i*. Eq (11) follows from P[A∩B]≤min(P[A],P[B]) while Eq (12) follows from the fact that the minimum of a set of numbers is less than their average. Summing over all nodes, the upper bound on the expected number of node failures in the gird (*N*_*f*_) is given by:

$$\begin{array}{cc} & N_{f}=\sum_{i=1}^{N}\left(1-\lambda^{V}\left(i\right)\right)\\ \Rightarrow & N_{f}\leq p_{0}N+\left(1-p_{0}\right)\sum_{i=1}^{N}\sum_{j:(ij)\in E}\frac{1-\lambda^{E}\left(ij\right)}{d_{i}} \end{array}$$ (13)

Note that *N*_*f*_ depends on λ^*E*^. Previously Eq (8) gave an upper bound on λ. Here we require an upper bound on 1 − λ^*E*^. For that we use a similar analysis as Eqs (10–12) and derive expressions for (1 − λ^*E*^(*ij*)) for failure probabilities in the modified graph.

$$\begin{array}{cc} 1-\lambda^{E}\left(ij\right) & =p_{0}+\left(1-p_{0}\right)P\left[\underset{k:(jk)\in E,k\neq i}{⋂}{\left[B^{E}\left(jk\right)\right]}^{c}\right] \end{array}$$ (14)

$$\begin{array}{cc} & \leq p_{0}+\left(1-p_{0}\right)\sum_{k:(jk)\in E,k\neq i}\frac{1-\lambda^{E}\left(jk\right)}{d_{ij}} \end{array}$$ (15)

where *d*_*ij*_ is the number of neighbors of node *v*_*ij*_ in modified graph $G^{E}$. Writing it in vector form, we get

$$\begin{array}{c} 1-\lambda^{E}=p_{0}{\left(I_{2|E|}-\left(1-p_{0}\right)D_{E}^{-1}A_{E}\right)}^{-1} \end{array}$$ (16)

where $I_{2|E|}$ is the identity matrix of dimension 2|*E*| (number of nodes in $G^{E}$). *D*_*E*_ is the diagonal matrix of node degrees (*d*_*ij*_) in $G^{E}$, while *A*_*E*_ is its adjacency matrix. We can now compute the **upper bound** on node failures as summarized in the following theorem.

**Theorem 3** *Consider grid graph* G *with edge set E and modified directed graph* $G^{E}$. *Let A*_*E*_ *and D*_*E*_ *respectively be the adjacency and degree matrices of* $G^{E}$. *The expected number of nodal failures N*_*f*_ *after a natural disaster that induces an initial failure probability p*_0_ *is upper bounded by the right side of* Eq (13), *where* **1** − λ^*E*^ *is given by* Eq (16).

To demonstrate the performance of our bounds, we present simulations of random failures on known power grid graphs.

### Comparison of bounds through simulations

**Fig 4:**
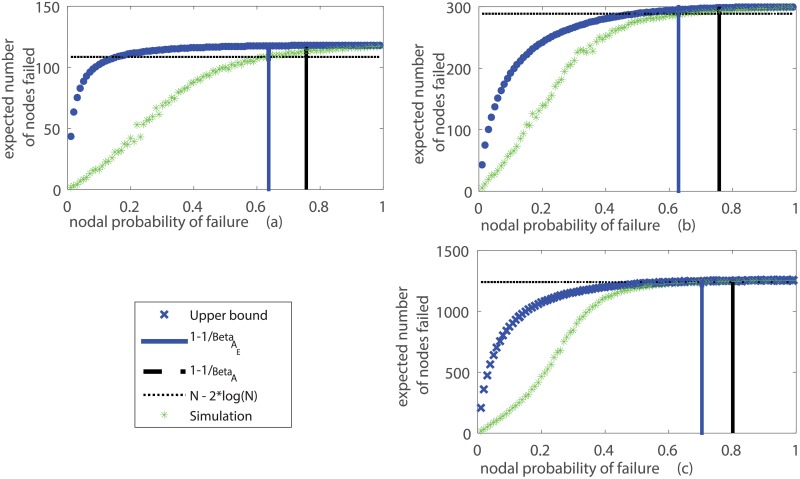
Upper bounds for number of failed nodes and bounds on *p*_0_ in (a) IEEE 118 bus system (b) IEEE 300 bus system (c) UCTE power grid.

The performances of the upper bound on network failure over all values of *p*_0_ and the two upper bounds on critical value of *p*_0_ beyond which the network disintegrates are shown through simulations on the IEEE 118 and 300 bus test systems [35] in Fig 4(a) and 4(b) respectively. Subsequently we also consider publicly available power grid topologies pertaining to the grid under the Union for Coordination of Transmission of Electricity (UCTE) in Europe. The power grid of the UCTE has 1254 buses and 1811 lines [41, 42]. Failure propagation simulation and determined bounds for this network are shown in Fig 4(c). All simulations have been carried out in Matlab 2008 on a windows 8 machine.

In all the power grid cases, we consider the grid to have fragmented if the size of the largest connected component of surviving nodes is less than 2 log *N*, where *N* is the initial size of the network. This follows from the usage of logarithm of the original network size as a popular scale in graph percolation to signify total fragmentation [43]. It is true that for operational paradigms in power grids where additional information of nodal type and control architectures are available, percolation based notions of fragmentation may not present the entire picture. However we restrict ourselves to such bounds as we are strictly concerned with topological vulnerabilities in this paper and only analyze the affect of grid structure. In future work, we plan to merge topological notions of failure with operational and power flow based features.

In Fig 4(a)–4(c), it can be clearly observed that the novel second upper bound ($1-1/\beta_{A_{E}}$) on the threshold on *p*_0_ (initial probability of failure) derived from the modified graph is lower and hence tighter than the first upper bound (1 − 1/*β*_*A*_) derived using the original graph. Next we discuss the upper bound on number of grid failures given by Theorem 3. Note that the upper bounds given by Eqs (13) and (16) is tighter for higher values of *p*_0_ compared to smaller values. The gaps between the simulated number of failures and the upper bound arise due to the presence of loops/cycles in the power grid graphs. These loops creates multiple paths between nodes and enhances grid robustness to fragmentation after the disaster. However the connectivity through the multiple paths are correlated and hence the inequalities described in Eqs (6) and (15) are weaker. In the absence of cycles, the grid graph is radial/tree-like where unique paths exist between any two nodes or between a node and the largest component. In this setting the upper bound given by Eqs (13) and (16) closely reflects the actual number of failures and will be tight.

The crucial advantage of the modified graph based analysis is the non-trivial upper bound on number of failures for values of *p*_0_ below the threshold that are not captured by the original graph and in random graph based prior work. This is in addition to the tighter bound on threshold for network breakdown. As mentioned in the Introduction, one of the chief advantages of using topology information in determining measures of grid failure is in understanding vulnerable grid components that can help in securing the grid against adversarial attacks. This is particularly useful compared to random graph based models on power grids where specific nodes’ effects on the grid vulnerability cannot be directly measured.

In the next section, we present algorithms to determine critical transmission lines that may be attacked by an adversary interested in weakening the grid resilience to natural disasters.

## Critical lines for adversarial attack on grid resilience

We consider an adversary that aims to maximally weaken the grid structure to make it more vulnerable to failures during natural disasters. The adversary does so by attacking and removing a fixed number (*k*_*max*_) of transmission lines in the grid. As mentioned in prior sections, relations involving the eigenvalues of adjacency matrix of the grid graph or the modified graph provide upper bounds on the probability of failure beyond which grid connectivity diminishes greatly. *Decreasing* these upper bounds will enhance the vulnerability of the network to disasters. In the remainder of this section, we focus on the first upper bound $\left(1-1/\beta_{A_{G}}\right)$ given in Theorem 1. Techniques based on the modified graph in Eq (9) will be the focus of a future work. Let normalized eigenvector *u*_1_ correspond to the largest eigenvalue $\beta_{A_{G}}$ of adjacency matrix $A_{G}$. By definition

$$\begin{array}{c} \max_{{∥x∥}_{2}=1}x^{T}A_{G}x=u_{1}^{T}A_{G}u_{1}=\beta_{A_{G}} \end{array}$$ (17)

The Perron-Frobenius theorem [44] states that eigenvector *u*_1_ is a positive vector. From Eq (17), it is thus clear that removing edges from graph G (or deleting 1s from $A_{G}$) leads to a reduction in the magnitude of its largest eigenvalue. Hence, we formulate the adversary’s objective as follows.

**Attack Problem**: Identify and damage *k*_*max*_ edges in the graph to minimize $\beta_{A_{G}}$, where $\beta_{A_{G}}$ is the largest eigenvalue of the adjacency matrix $A_{G}$.

This is a known NP-hard problem. Here, we present three approximate techniques to determine the critical lines that will be included in the adversary’s target set.

### Eigen-Perturbation based attack

In this attack scheme, we use perturbation analysis [44] to approximate the change in eigenvalues of adjacency matrix $A_{G}$ following removal of edges. Let the new adjacency matrix of the grid after removal of edges be given by $A_{G}-\Delta A_{G}$. The change in adjacency matrix $\Delta A_{G}$ has the following structure:

$$\begin{array}{c} \Delta A_{G}\left(i,j\right)=\{\begin{array}{cc} 1 & \;\;\text{if}\;\text{edge}\;(ij)\in E\;\text{is}\;\text{removed},\\ 0 & \;\;\text{otherwise,} \end{array} \end{array}$$ (18)

Let the largest eigenvalue of new adjacency matrix be $\beta_{A_{G}}^{\Delta }$. From Eq (17), we have

$$\begin{array}{cc} & \beta_{A_{G}}^{\Delta }=\max_{{∥x∥}_{2}=1}x^{T}\left(A_{G}-\Delta A_{G}\right)x\\ \Rightarrow & \beta_{A_{G}}^{\Delta }\geq {u_{1}}^{T}\left(A_{G}-\Delta A_{G}\right)u_{1}=\beta_{A_{G}}-{u_{1}}^{T}\Delta A_{G}u_{1}\\ \Rightarrow & \beta_{A_{G}}-\beta_{A_{G}}^{\Delta }\leq {u_{1}}^{T}\Delta A_{G}u_{1}\\ \Rightarrow & \Delta \beta_{A_{G}}\approx \sum_{\text{removed}\;(ij)}2u_{1}\left(i\right)u_{1}\left(j\right)\;\;\left(\text{using}\;\text{Eq}\;\;\left(18\right)\right) \end{array}$$ (19)

where $\Delta \beta_{A_{G}}=\beta_{A_{G}}-\beta_{A_{G}}^{\Delta }$ denotes the change in the maximum eigen-value.

**The optimal transmission lines in the attack are then determined by maximizing the linear expression in Eq (19).** Next, we use this to design a modified attack scheme.

### Iterative Eigen-Perturbation based attack

In this scheme, the adversary does not select all *k*_*max*_ lines together by maximizing Eq (19). Instead, it selects them iteratively, one at a time. In each iteration, it computes *u*_1_, the eigenvector corresponding to the largest eigenvalue, removes line to maximize Eq (19) and refines the adjacency matrix. As shown later, the iterative scheme provides far greater reduction in grid resilience than selecting the *k*_*max*_ lines all at once, though it leads to an increase in computational complexity.

**Complexity:** The computation of the eigenvector *u*_1_ takes *O*(*N*^3^) steps via Singular Value Decomposition (SVD) of $A_{G}$ [45]. Given that the maximization of Eq (19) takes *k*_*max*_|*E*| steps which is less than *O*(*N*^3^), computing the *k*_*max*_ critical lines by the first non-iterative approach takes *O*(*N*^3^). On the other hand, the iterative scheme needs *k*_*max*_ SVD computations and hence has a complexity of *O*(*k*_*max*_ *N*^3^). Finally, we describe another technique for attack design that depends on trace minimization.

### Trace minimization based attack

The trace of a square matrix refers to the sum of its diagonal elements and is equal to the sum of its eigenvalues [44]. Consider the 2*r*^*th*^ power of the adjacency matrix $A_{G}$, where 2*r* is an even positive integer. The eigenvalues of $A_{G}^{2r}$ are the 2*r*^*th*^ powers of the eigenvalues ($\beta_{A_{G}}=\beta_{1}>\beta_{2},\ldots \geq \beta_{N}$) of $A_{G}$. We thus have the following relation for its trace

$$\begin{array}{cc} & trace\left(A_{G}^{2r}\right)=\sum_{i=1}^{N}\beta_{i}^{2r}=\beta_{A_{G}}^{2r}\left(1+{\left(\frac{\beta_{2}}{\beta_{A_{G}}}\right)}^{2r}+\ldots +{\left(\frac{\beta_{N}}{\beta_{A_{G}}}\right)}^{2r}\right) \end{array}$$ (20)

$$\begin{array}{cc} & \Rightarrow \;\lim_{r\to \infty }trace\left(A_{G}^{2r}\right)/\beta_{A_{G}}^{2r}\approx 1 \end{array}$$ (21)

where $\beta_{A_{G}}=\beta_{1}$ is the largest eigenvalue of the adjacency matrix. Eq (21) follows as $|\frac{\beta_{i}}{\beta_{A_{G}}}|<1\forall 2\leq i\leq N$. Thus, for large values of *r*, the largest eigenvalue and trace of the 2*r*^*th*^ power of $A_{G}$ are approximately equal. In this attack design, we thus remove lines to reduce the trace of $A_{G}^{2r}$ instead of the largest eigenvalue $\beta_{A_{G}}$ or its higher power $\beta_{A_{G}}^{2r}$. Optimal minimization of the trace of $A_{G}^{2r}$ is computationally hard as well, however it has certain advantages as discussed in the next result.

**Theorem 4** *The trace of* $A_{G}^{2r}$, *where* $A_{G}$ *is the adjacency matrix of grid graph* G *is a supermodular function of the constituent edges in the graph*.

**Note:** A real-valued function *f* defined over set *S* is *supermodular* [46] if *f*(*A* ⋃ *C*) − *f*(*A*) ≥ *f*(*B* ⋃ *C*) − *f*(*B*) for *B* ⊂ *A* and *A*, *B*, *C* are subsets of *S*. In other words, the returns due to addition of *C* are not diminishing.

**Proof:** Let a cycle of length 2*r* in $A_{G}$ refer to a graph path with 2*r* hops (repetition allowed) that begins and ends at the same node. Note that the *i*^*th*^ diagonal element in $A_{G}^{2r}$ is equal to the number of cycles of length 2*r* that begin and end at node *i*. This can be shown by direct checks or by mathematical induction. Thus, the trace of $A_{G}^{2r}$ is given by the total number of cycles of length 2*r* formed on all nodes in the grid graph. To show that trace of $A_{G}^{2r}$ is a supermodular function, we consider edge sets *A*, *B* = *A* ⋃ {(*lm*)}, *C* = {(*ij*)} for edges (*lm*), (*ij*) ∉ *A*. We argue that the increase in the number of cycles of length 2*r* in edge set *A* is less than the increase observed in set *B* due to addition of edge (*ij*). This is true as presence of edge (*lm*) in *B* ∪ *C* produces cycles having both edges (*lm*) and (*ij*) that cannot be formed in *A* ∪ *C*. The trace of $A_{G}^{2r}$ is thus a supermodular function of the graph edges.

It is a known that greedy minimization of a supermodular function is equivalent to greedy maximization of a submodular function and is provably at least 1 − 1/*e* (≈ 63%) close to the optimal solution [46]. Thus, the adversary’s attack policy in this scheme is **to greedily remove *k*_*max*_ edges that minimizes the trace of 2*r*^*th*^ power of the adjacency matrix of the grid graph.**

**Complexity:** The 2*r*^*th*^ power of the symmetric adjacency matrix is computed efficiently using Singular Value Decomposition (SVD) as $A_{G}^{2r}=U\beta_{A_{G}}^{2r}U^{T}$ where columns of *U* are the eigenvectors and $\beta_{A_{G}}^{2r}$ is the diagonal matrix with 2*r*^*th*^ powers of the eigenvalues. Note that matrix multiplication and SVD are computed in *O*(*N*^3^) while computing $\beta_{A_{G}}^{2r}$ takes complexity *O*(*N* log *r*). Since we greedily minimize the trace, the selection of one edge takes *O*(|*E*|(*N*^3^ + *N* log *r*)). The overall complexity of computing *k*_*max*_ optimal edges by this scheme is thus *O*(*k*_*max*_|*E*|(*N*^3^ + *N* log *r*)). This expression implies that increasing *r* to improve the accuracy of this approach will at most lead to a logarithmic increase in the complexity.

**Resilience:** From the grid controller’s perspective, these techniques can be used to determine the critical transmission lines for enhancing security and reinforcement to prevent adversarial manipulation aimed at disrupting grid resilience to natural disasters. In the next section, we look at the performance of these approaches as an adversarial tool.

## Simulation results of adversarial attacks

**Fig 5:**
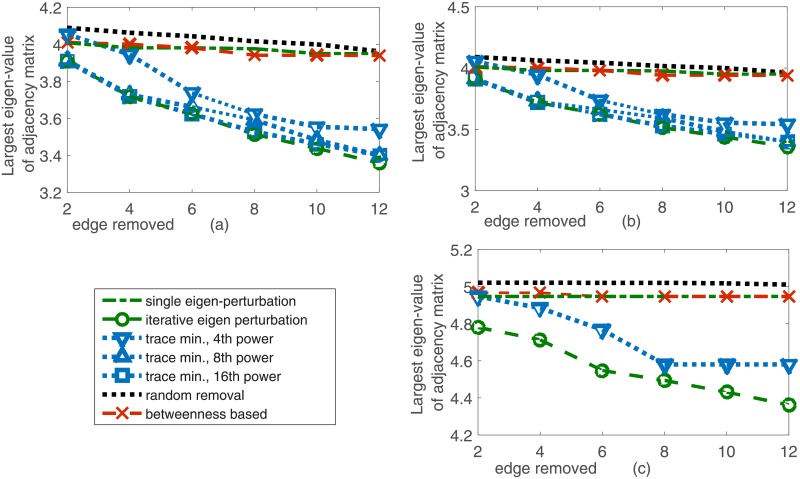
Comparison of adversarial schemes to reduce maximum eigenvalue of grid adjacency matrix in (a) IEEE 118 bus test system (b) IEEE 300 bus test system (c) UCTE power grid.

We consider all approaches (eigen perturbation, iterative eigen perturbation and trace minimization) for determining the optimal *k*_*max*_ edges to minimize the largest eigenvalue of the adjacency matrix of the grid graph and thereby reduce the resilience of the grid to natural disasters. Note that each of these methods use global properties of the network in determining the most vulnerable edges. To demonstrate the improvements from our schemes, we compare them with two alternate schemes: one where an adversary removes edges randomly, and another where an adversary removes edges in the decreasing order of their betweenness centralities suggested in prior work [14]. We plot our results for the IEEE 118 and 300 bus test systems and the UCTE power grid network in Fig 5(a), 5(b) and 5(c) respectively. Note that both algorithms outperform random and betweenness based attacks [19, 31] to reduce the eigenvalues. It can also be noted that iterative eigen perturbation reduces the largest eigenvalue further than edge removal based on a single perturbation computation as mentioned in the previous section. Further, it can be observed from Fig 5(a) and 5(b) that increasing the value of 2*r*, the power of the adjacency matrix, leads to an improvement in the trace minimization based scheme as it approximates the largest eigenvalue better as noted in Eq (21). These plots highlight the benefit derived from utilizing the structural information of the network in studying the vulnerable components of the grid.

## Limitations and extensions

In this section, we discuss simplifications in our model in comparison to other techniques and present a path forward to its incorporation into general grid resilience research. As the primary objective of the work is in understanding topological effects on grid vulnerability, we limit our study to connectivity based failures where we show improved performance over general random graph based prior work. It needs to be mentioned that studying size of largest connected component in a grid is realistic when looking at distributed placement of similar controllers or generators throughout the network. In the dynamic regime, the larger the connected component is, the greater is its ability to withstand fluctuations in voltage and frequency. However including dynamics models of power flows in the analysis is important. Note that cascading failures in the grid are complex processes that begin at slower time-scales with overloading of lines etc. and eventually at faster time-scales due to frequency and voltage based automatic tripping of different components. At different scales, different models for network flow are applicable and hence a single method is not sufficient to understand vulnerability to failures. Further different components (generators/loads) may have differing failure models. One potential way of extending our methodology to multiple component types would include keeping different probabilities of nodal failures and dependence on neighbors.

In future extensions, nodal types (generator/load/distributor) and system constraints (line limits/nodal voltage limits/power capacities) should be included to in the topological failure model, though analytic formulae for thresholds may be harder to derive. Further if knowledge of node type is available, grid failures can be studied at group levels (sub-networks) that can self-sustain if disconnected. It is unlikely that a single method of vulnerability analysis can approximate the failure propagation in the full-scale power grid model. Hence a judicious combination of topological models, static power flow based and grid dynamics based models should be utilized to determine optimal actions and reinforcements to enable the protection of the grid.

## Conclusion

We analyze topological vulnerability of power grids to probabilistic failures introduced by natural disasters in this paper. We analyze the evolving failure process that originates at nodes with initial failures. Based on the adjacency matrix of the grid, we present two bounds on the critical initial probability of node failures beyond which the grid fragments. We also derive a new non-trivial upper bound on the expected number of total failures after the disaster. We present the performance of our derived bounds on two IEEE test cases and a real grid data set. Finally, we discuss adversarial attacks on the power grid aimed at damaging transmission lines to minimize the grid’s resilience to natural disasters. We develop approximate algorithms to identify the critical lines that will enable such adversarial attacks—the first two based on perturbation analysis of the eigenvalues of the adjacency matrix, and the third algorithm based on minimization of the trace of a higher power of the adjacency matrix. We analyze the complexity of both algorithms and demonstrate their performance against random and centrality based attacks studied in literature through simulations.

Moving forward, we plan to generalize our failure models with respect to diversity of nodes and initial probability of failures. Further we plan to incorporate our model with existing work as mentioned in the preceding section.

In future work, we plan to build upon the techniques developed in this paper to incorporate more realistic features of grid operations.
